# Gut microbiota phenotypes of obesity

**DOI:** 10.1038/s41522-019-0091-8

**Published:** 2019-07-01

**Authors:** Maggie A. Stanislawski, Dana Dabelea, Leslie A. Lange, Brandie D. Wagner, Catherine A. Lozupone

**Affiliations:** 1 Department of Epidemiology, Colorado School of Public Health, Aurora, CO USA; 2 Lifecourse Epidemiology of Adiposity and Diabetes (LEAD) Center, Aurora, CO USA; 3 Department of Pediatrics, University of Colorado School of Medicine, Aurora, CO USA; 4 Department of Medicine, Division of Biomedical Informatics and Personalized Medicine, University of Colorado School of Medicine, Aurora, CO USA; 5 Department of Biostatistics and Informatics, Colorado School of Public Health, Aurora, CO USA

**Keywords:** Microbiota, Microbial communities

## Abstract

Obesity is a disease with a complex etiology and variable prevalence across different populations. While several studies have reported gut microbiota composition differences associated with obesity in humans, there has been a lack of consistency in the nature of the reported changes; it has been difficult to determine whether methodological differences between studies, underlying differences in the populations studied, or other factors are responsible for this discordance. Here we use 16 S rRNA data from previously published studies to explore how the gut microbiota-obesity relationship varies across heterogeneous Western populations, focusing mainly on the relationship between (1) alpha diversity and (2) *Prevotella* relative abundance with BMI. We provide evidence that the relationship between lower alpha diversity and higher BMI may be most consistent in non-Hispanic white (NHW) populations and/or those with high socioeconomic status, while the relationship between higher *Prevotella* relative abundance and BMI may be stronger among black and Hispanic populations. We further examine how diet may impact these relationships. This work suggests that gut microbiota phenotypes of obesity may differ with race/ethnicity or its correlates, such as dietary components or socioeconomic status. However, microbiome cohorts are often too small to study complex interaction effects and non-white individuals are greatly underrepresented, creating substantial challenges to understanding population-level patterns in the microbiome-obesity relationship. Further study of how population heterogeneity influences the relationship between the gut microbiota and obesity is warranted.

## Introduction

Obesity has reached epidemic proportions in Western populations.^1^ Studies aiming to determine the cause of this precipitous increase are complicated by the mixed etiology and manifestations of the disease. Individuals vary greatly in their propensity toward obesity and in the extent to which obesity is associated with metabolic complications such as hyperlipidemia, hypertension, glucose intolerance and diabetes, or other adverse health conditions.^2^ Obesity is influenced by diet, behavior, and other environmental as well as genetic factors, and susceptibility to obesity differs by sex, age, race, ethnicity, and socioeconomic status.^1^ Racial/ethnic differences in obesity prevalence are especially high for women, with Non-Hispanic black and Hispanic women having much higher prevalence compared to non-Hispanic whites (NHW).^1,3^

Many studies have reported differences in microbiota composition between obese and lean humans.^2,4,5^ Mouse experiments have provided compelling evidence that these differences are not merely correlational, and many potential mechanisms for these actions have been elucidated.^6–9^ Studies of the nature of compositional differences that occur in obese versus lean humans, however, have been difficult to interpret because the results are often not in agreement.^5,10^ One property that has been reported in multiple studies of obesity is reduced richness of microbes or their genes.^4,11–13^ However, although individuals with reduced richness are more often obese, low richness is not observed in the majority of obese individuals,^11^ indicating that low richness only has the potential to be important in a subset of obese individuals. The specific taxa reported to differ with obesity have varied across studies. For instance, the ratio of Bacteroidetes to Firmicutes in obese versus lean humans has been reported to decrease to increase or to not change at all.^5^ Similarly, one small study found that morbidly obese individuals had increased relative abundance of *Prevotella*,^14^ and *Prevotella* relative abundance positively correlated with BMI in a cohort of HIV positive individuals and controls in Mexico City,^15^ but *Prevotella* dominated communities were explicitly found to not correlate with Body Mass Index (BMI) in the Old Order Amish.^2^ These inconsistencies may be influenced by methodological differences between studies,^16^ by differences in composition that are not readily captured by the current technologies, or they may reflect that genetic, environmental, and/or lifestyle heterogeneity across the surveyed populations has an influence on the nature of the microbial associations that occur with obesity and related metabolic conditions.^5^ These influences are poorly understood both in terms of effects on the gut microbiota composition overall and in the context of the disease-specific relationships.^17,18^

In this study, we explore the hypothesis that population heterogeneity, particularly race/ethnicity, may impact the gut microbiota-obesity relationship. This hypothesis was motivated by a meta-analysis involving data from two previously published studies that included 16 S rRNA sequencing of the fecal microbiome: (1) a cohort of 152 obese and lean female adult twins and their mothers from Missouri^4^ (*N* = 75 NHW / 77 black; we refer to this study as “Obese Twins”), and (2) a cohort of lean individuals across the age spectrum from the US and agrarian cultures in Malawi and the Amazonas State of Venezuela (we refer to this study as “Global Gut,” abbreviated GG).^19^ We explored the patterns uncovered in this meta-analysis using three additional cohorts that included NHW, black and/or Hispanic individuals with a wide range of BMI (1) a healthy subset of individuals from the American Gut cohort (AG; *N* = 5035; 4784 NHW/188 Hispanic/63 black),^20^ (2) a cohort of teenagers from the Exploring Perinatal Outcomes among Children study (EPOCH; *N* = 102; 59 NHW/43 Hispanic),^21^ and (3) a cohort of HIV positive and negative Mexican individuals (referred to as the “Mexico City” cohort; *N* = 42 Hispanic).^15^ We examine the many correlates of race/ethnicity, including the prevalence and severity of obesity, diet, smoking status, and socioeconomic status, and we attempt to understand how these inter-related factors may impact the relationship between the gut microbiota and obesity.

## Results

### Gut microbiota subtypes of obesity observed in Obese Twins

We first observed that there may be distinct gut microbiota phenotypes associated with obesity while performing a meta-analysis that combined 16 S ribosomal RNA (rRNA) data from Obese Twins^4^ and the GG study^19^ (Supplementary Fig. 1). The GG dataset is useful for meta-analysis because it includes fecal samples from individuals across the two most dramatic microbiota-composition gradients observed across the human population:^19,22^ (1) microbiomes cluster by age, with infants having a low-diversity microbiota with dramatically different composition from adults, and (2) microbiomes cluster by culture, with individuals from agrarian cultures having a *Prevotella*-rich/Bacteroides poor microbiota that differs substantially from individuals from the US and Europe.^19,22^ We applied Principal Coordinates Analysis (PCoA) to an unweighted UniFrac distance matrix of the combined data.^23^ We note that we used previously unpublished sequence data from the Obese Twins and GG studies that were processed together using the same methods (see Methods for more detail). The PCoA analysis produced clustering from old to young age from left to right (Supplementary Fig. 1a; *p*-value for association with age ≤0.01), and from Western to Agrarian culture from top to bottom (Supplementary Fig. 1b). The first principal coordinates axis (PC1) correlated negatively with alpha diversity (*p*-value < 0.01 for Phylogenetic Diversity and Shannon diversity index). PC2 correlated inversely with relative abundance of the genus *Prevotella* (*p*-value < 0.01), consistent with high *Prevotella* being observed in agrarian populations.^19,24^

### Race correlates with gut microbiota subtypes in Obese Twins

Obese individuals showed greater spread across both the age and culture axes of variation than either lean or overweight individuals from Obese Twins (Supplementary Fig. 1c; *p*-value < 0.001). As seen in many studies, many obese individuals clustered with lean individuals,^11^ but there were two notable clusters of outliers of obese individuals, one spreading towards infants/lower alpha diversity on PC1, and the other spreading towards the *Prevotella*-rich/*Bacteroides* poor communities of agrarian cultures on PC2. We noted that the obese outliers on PC1 tended to be white, while those on PC2 tended to be black (Supplementary Fig. 1c–e). Comparing individuals by obesity status and stratifying by race (Table 1), we noted that among whites, the average alpha diversity was lower among obese individuals, but not significantly so. Among blacks, average alpha diversity was significantly higher among obese individuals, which was surprising given the prior literature showing an association between lower alpha diversity and obesity.^4,11–13^

**Table 1:** Characteristics of the individuals in the Obese Twins cohort overall and by obesity status, stratified by race

	All	White			Black		
		Non-obese	Obese	*p*-value	Non-obese	Obese	*p*-value
Number of individuals	152	34	41		15	62	
Number of samples	270	62	71		21	116	
Age	34.0 (12.2)	32.7 (11.7)	35.9 (13.2)	0.266	37.7 (14.6)	32.6 (11.1)	0.136
Hispanic ethnicity				0.695			0.092
No	134 (88.2)	0 (0.0)	0 (0.0)		11 (73.3)	55 (88.7)	
Yes	1 (0.7)	0 (0.0)	0 (0.0)		1 (6.7)	0 (0.0)	
Unknown	17 (11.2)	4 (11.8)	3 (7.3)		7 (11.3)	3 (7.3)	0.736
Average BMI	34.8 (10.4)	22.8 (2.8)	39.2 (5.9)	<0.001	24.3 (3.5)	41.1 (8.7)	<0.001
BMI category				<0.001			<0.001
Lean	34 (22.4)	26 (76.5)	0 (0.0)		8 (53.3)	0 (0.0)	
Overweight	15 (9.9)	8 (23.5)	0 (0.0)		7 (46.7)	0 (0.0)	
Obese (Total)	103 (67.8)	0 (0.0)	41 (100.0)		0 (0.0)	62 (100.0)	
Obese category I (30 < BMI < 35)	24 (15.8)	0 (0.0)	11 (26.8)		0 (0.0)	13 (21.0)	
Obese category II (35 < BMI < 40)	33 (21.7)	0 (0.0)	13 (31.7)		0 (0.0)	20 (32.3)	
Obese category III + (BMI > 40)	46 (30.3)	0 (0.0)	17 (41.5)		0 (0.0)	29 (46.8)	
Twin (versus mother of twin)	106 (69.7)	26 (76.5)	26 (63.4)	0.332	8 (53.3)	46 (74.2)	0.204
Monozygotic twin	60 (56.6)	17 (65.4)	13 (50.0)	0.4	7 (87.5)	23 (50.0)	0.113
*Smoking status*							
Smoker	39 (25.7)	8 (23.5)	13 (31.7)	0.392	2 (13.3)	16 (25.8)	0.353
Non-smoker	107 (70.4)	23 (67.6)	27 (65.9)		12 (80.0)	45 (72.6)	
Unknown	6 (3.9)	3 (8.8)	1 (2.4)		1 (6.7)	1 (1.6)	
*Diet*							
Non-missing dietary information	144 (94.7)	31 (91.2)	38 (92.7)	>0.99	14 (93.3)	61 (98.4)	0.842
Kilocalories	1489 (665.8)	1171 (479.5)	1567 (604.1)	0.004	1291 (548.3)	1647 (748.9)	0.098
Protein (%)	15.0 (3.4)	15.8 (3.3)	15.4 (2.0)	0.614	15.4 (4.3)	14.30 (3.45)	0.305
Meat servings	1.8 (1.1)	1.2 (0.6)	1.8 (0.8)	0.001	1.4 (0.9)	2.1 (1.3)	0.055
Fat (%)	38.7 (6.9)	38.4 (8.3)	41.1 (7.1)	0.154	35.3 (6.3)	38.2 (5.7)	0.09
Saturated fat (%)	0.1 (0.03)	0.14 (0.03)	0.14 (0.03)	0.224	0.12 (0.03)	0.12 (0.02)	0.412
Carbohydrates (%)	46.4 (8.6)	43.4 (9.3)	43.8 (7.9)	0.836	51.6 (9.0)	48.3 (7.5)	0.163
Sweets (%)	14.2 (9.8)	11.6 (9.2)	14.4 (9.8)	0.236	11.7 (9.9)	16 (9.9)	0.145
Fiber (%)	0.03 (0.02)	0.03 (0.02)	0.03 (0.01)	0.66	0.05 (0.02)	0.03 (0.01)	<0.001
Fiber from grains (%)	0.01 (0.01)	0.01 (0.01)	0.01 (0.01)	0.363	0.01 (0.01)	0.01 (0.00)	0.814
Fiber from vegetables (%)	0.02 (0.01)	0.02 (0.01)	0.02 (0.01)	0.921	0.03 (0.03)	0.02 (0.01)	0.001
*Alpha diversity (mean of samples)*							
Observed species	275.6 (39.9)	283 (28.9)	274 (42.1)	0.318	249.7 (39.8)	278.7 (41.8)	0.021
Shannon diversity index	6.7 (0.5)	6.8 (0.4)	6.7 (0.5)	0.442	6.3 (0.6)	6.7 (0.6)	0.035
Faith’s PD	3.2 (0.7)	3.3 (0.6)	3.2 (0.8)	0.367	2.9 (0.5)	3.2 (0.8)	0.161

To further examine the relationship between these PC axes, obesity status, and race, we used adjusted hierarchical linear regression models of BMI versus the PC axes by racial group (Supplementary Fig. 2a–b). PC1 was associated with higher BMI for white (β = 29.2 (3.9, 54.5); *p*-value = 0.025) but not black individuals (β = 13.6 (−25.2, 52.4); *p*-value = 0.485) from Obese Twins. PC2 showed a stronger pattern by race; PC2 correlated with lower BMI for black individuals (β = −23.5 (−46.6, −0.5); *p*-value = 0.045) and higher BMI for white individuals (β = 31.1 (4.1, 58.2); *p*-value = 0.02). While the PC2 axis correlated with the relative abundance of *Prevotella*, this differential pattern by racial group was much stronger with the PC2 axis than with *Prevotella* relative abundance alone (Supplementary Fig. 2c), indicating that this association is driven by a community type, which may be characterized by *Prevotella*, but reflects other gut microbiota as well.

### Gut microbiota-obesity relationship in other cohorts

It is difficult to know whether these differential relationships by race are driven by genetic ancestry or by the many environmental correlates of self-described race. In the Obese Twins study (Table 1), black individuals had higher prevalence and severity of obesity; there were many differences in diet by race (e.g., blacks had greater intake of meat and calories, higher proportion of carbohydrates and less of fat, saturated fat, and fiber from grains); and smoking prevalence was higher among whites. While we did not have information on socioeconomic status for this cohort, the larger Missouri Female Twin study has reported that whites had higher income, more education, greater family intactness and tended to live in less urban areas.^25^ It is challenging to untangle these complex inter-relationships, particularly given that most human studies of the gut microbiome have small sample sizes and are among predominantly NHW populations. However, we were able to explore variation in the gut microbiota-obesity relationship using 3 additional cohorts with at least some representation of other racial and ethnic groups (Tables S1–S3): the American Gut (AG) project (non-Hispanic white/NHW, Hispanic white, and black); the EPOCH study (NHW, Hispanic white) and a cohort from Mexico City of HIV-positive individuals and controls (Hispanic white). These studies likewise showed marked differences in obesity prevalence and severity, diet and socioeconomic status by race/ethnicity.

We first examined predictors of the overall gut microbiota composition in each of these studies using permutational ANOVA. Overweight/obese status, race, sex, and smoking status were generally associated with the gut microbiota composition across these studies (Supplementary Table 4). Both the Obese Twins and AG showed a significant interaction between race/ethnicity and overweight/obese status.

We also investigated whether there was variation in the gut microbiota-obesity relationship in these studies by race/ethnicity, as seen in Obese Twins. Since meta analyses of these other studies with GG would involve a study effect (i.e., the sequences were produced by different labs using different protocols such as PCR primers and DNA extraction techniques), rather than using PC axes in analyses (which did not correspond as directly with alpha diversity and culture), we explicitly examined the association between (1) alpha diversity and (2) *Prevotella* relative abundance with BMI in each study. These studies showed some corroboration that there may be differences in the relationship between the gut microbiota and obesity by race/ethnicity or its correlates.

### Inconsistencies in the alpha diversity-BMI relationship

**Fig. 1:**
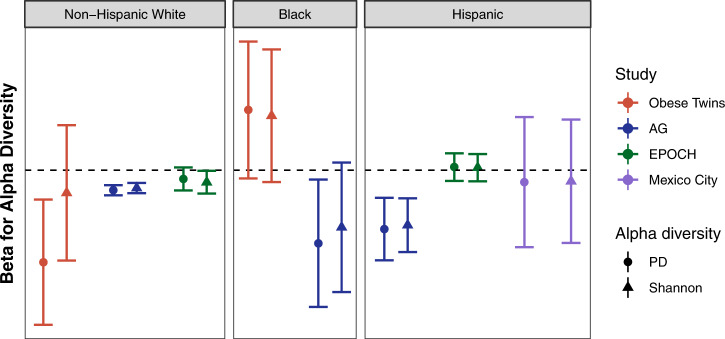
Results of adjusted regression models of BMI as a function of alpha diversity. These plots show the regression β estimates (and 95% confidence intervals) for phylogenetic diversity and Shannon diversity index. The dotted line shows 0; estimates significantly below the line indicate that lower alpha diversity is associated with higher BMI and vice versa. This plot suggests that the association often reported in the literature between lower alpha diversity and obesity may be most consistent among Non-Hispanic white populations (left) and/or among relatively healthy populations of high socioeconomic status, such as AG (shown in blue)

To explore whether the observation of decreased alpha diversity with obesity varied by race, we used linear regression models of BMI as a function of alpha diversity (phylogenetic diversity and Shannon diversity index) by racial group, controlling for potential confounding variables that varied with each cohort (see Methods). Interestingly, the association between low alpha diversity and increased BMI, which has been reported previously in several studies, was most consistent across studies among NHWs and most consistent across racial/ethnic groups in AG, which is a generally healthy cohort with moderate BMI and high socioeconomic status^20^ (Supplementary Table 1, Fig. 1).

**Fig. 2:**
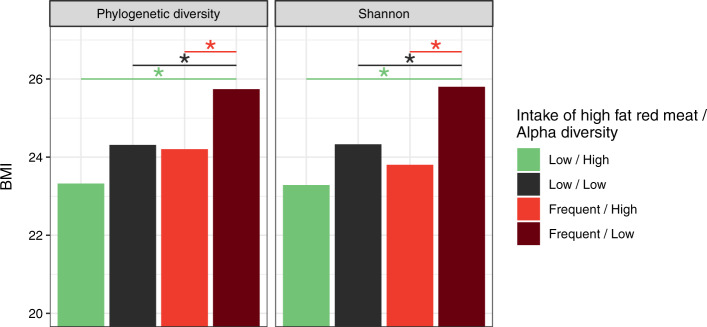
Estimated BMI by high fat red meat intake and alpha diversity. The relationship between alpha diversity and BMI (Fig. 1) in AG differed according to dietary intake of “high fat red meat.” No dietary interactions (fat, meat, or fiber) with alpha diversity were found in the other cohorts examined. This plot shows the estimated BMI from adjusted regression models by categories of intake of high fat red meat intake and alpha diversity (Phylogenetic and Shannon diversity): frequent (≥3 times per week) or low intake of high fat red meat and high (top quartile) or low-moderate alpha diversity. Those with frequent high fat red meat intake and low alpha diversity had significantly higher BMI than all other groups

We tested interactions between alpha diversity and dietary components (fiber, meat, and fat intake), which were non-significant in Obese Twins and EPOCH (dietary intake was not available for Mexico City). In AG, there was a significant interaction between alpha diversity and intake of “high fat red meat.” Individuals who consumed high fat red meat 3–5 times/week or more and had low-moderate alpha diversity (lower 3 quartiles) had the highest BMI, whereas those consumed high fat red meat less than three times/week and had high alpha diversity (top quartile) had the lowest BMI (Fig. 2).

### Inconsistencies in the Prevotella-BMI relationship

**Fig. 3:**
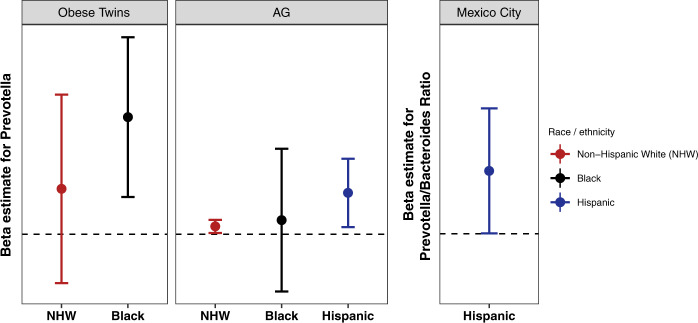
Results of adjusted regression models of BMI as a function of *Prevotella*. These plots show the regression β estimates (and 95% confidence intervals) for *Prevotella* (relative abundance in Obese Twins and AG; the ratio of *Prevotella* relative abundance to the sum of the relative abundance of *Prevotella* and *Bacteroides* in Mexico City; see Methods for details). The dotted line shows 0; estimates significantly above the line indicate that higher *Prevotella* is associated with higher BMI. This plot shows that the magnitude of the effect estimates for *Prevotella* is larger for blacks and Hispanics than for NHWs

**Fig. 4:**
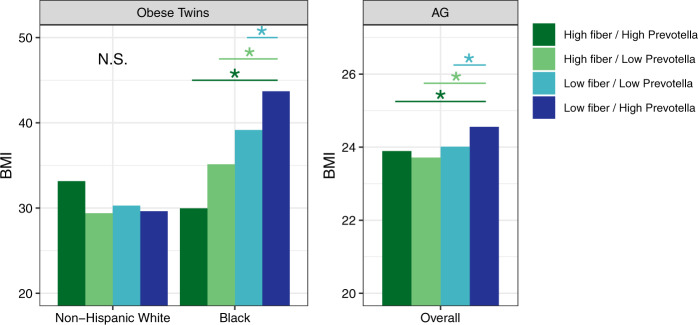
The relationship between *Prevotella* and BMI (Fig. 3) in Obese Twins and AG differed according to dietary intake of fiber. This plot shows the estimated BMI from adjusted regression models by categories of high/low fiber and *Prevotella* relative abundance, defined according to the different measures in each study (see Methods). The interaction between fiber and *Prevotella* was only apparent in blacks in Obese Twins; AG lacked power to examine these categories by race/ethnicity. In both studies, individuals with low fiber and high *Prevotella* had the highest BMI, and those with high fiber and *Prevotella* had significantly lower BMI

Next, we examined the relationship between *Prevotella* and BMI. We quantified *Prevotella* as both relative abundance and as the ratio of *Prevotella* to *Bacteroides* (see Methods); we selected the better predictor of BMI using adjusted R^2^ values from regression models. We saw an association between *Prevotella* and BMI in all cohorts except for EPOCH. We also found significant interactions between *Prevotella* and race/ethnicity, with the magnitude of the effect estimate for *Prevotella* increasing from NHWs to Blacks to Hispanics (Fig. 3). Since *Prevotella* has previously been associated with diet, specifically diets high in fiber and low in animal products/fat, and since there is evidence that *Prevotella* may interact with diet in relation to insulin sensitivity,^19,26,27^ we were curious as to whether there was an interaction between diet (fiber, meat, or fat intake) and *Prevotella* in relation to BMI. This was the case for fiber in Obese Twins and in AG. In Obese Twins, the interaction was only evident among blacks, who had generally higher BMI and substantially greater variation in BMI than whites. In AG, we did not have power to examine the interaction by race/ethnicity. Categorizing individuals according to low/high *Prevotella* and low/high fiber, individuals with high *Prevotella*/low fiber had the highest BMI and significantly higher than all other groups (Fig. 4).

Since prior evidence supports an association between smoking and lower alpha diversity, as well as with higher *Prevotella*,^28^ and smoking relates to obesity,^29^ we examined the possible role of smoking as a confounder or effect modifier in these relationships in Obese Twins and AG. All of the individuals from the EPOCH study were non-smokers, and the Mexico City study did not report smoking status. We saw no evidence of an association between smoking and lower alpha diversity (*p*-values non-significant with positive effect estimates); or between smoking and *Prevotella*. In Obese Twins, we saw no association between smoking and BMI, but smoking was correlated with higher BMI in AG (*p* = 0.02). We did not see evidence of interactions between smoking and alpha diversity or *Prevotella* in relation to BMI. Thus, we treated smoking as a potential confounder and adjusted for it in the regression models.

## Discussion

In this study, we found preliminary evidence across four studies that race/ethnicity, or its correlates, may be associated with distinct gut microbiota subtypes of obesity. In all of the studies included here with NHW individuals, there was evidence of a low alpha diversity subtype of obesity, and the association between lower alpha diversity and higher BMI was most consistent and strongest among NHWs and in AG, which is a cohort of predominantly healthy individuals with moderate BMI and high socioeconomic status. This relationship was notably less consistent among black and Hispanic individuals. We additionally noted a novel subtype of obesity in which the gut microbiota composition was less “Western”, more similar to that of individuals from agrarian cultures and high in *Prevotella*. While we did see at least some evidence of this gut microbiota phenotype of obesity in three of four studies examined, and some indication of an association with race/ethnicity, it would need to be validated in larger and more diverse cohorts. It is notable, however, that we found some evidence of variation of the gut microbiota-obesity relationship with race/ethnicity in four very different cohorts in terms of the population characteristics: adult twins and their mothers from Missouri, a heterogeneous cohort of healthy highly educated Americans from across the country, teenagers from Colorado, and a cohort of HIV positive and control adults from Mexico City.

These results raise many important questions about the role of race/ethnicity, ancestry, and genetics in the gut microbiota-obesity relationship, as well as the influence of lifestyle factors, such as diet, socioeconomic status, and smoking, all of which demand larger more racially diverse studies to thoroughly address. As obesity is known to have a genetic component and a link with microbiota composition, it stands to reason that genes may impact microbiota composition in a manner that in turn influences susceptibility to obesity. This notion was supported in a study of the microbiota of >1000 fecal samples obtained from twins in the UK, where a co-occurring group of heritable bacteria were found to be enriched in individuals with low BMI; amendment of an obesity-associated microbiota with the heritable lean-associated species *Christensenella minuta* resulted in reduced weight gain when transplanted into germ-free mice.^30^ The demonstrated link between heritable bacteria and protection from obesity supports that varied rates of obesity in different ethnic/racial populations may be driven by genetic polymorphisms in those populations that in turn result in varied selections for different microbiotas. Links between microbiota composition and ethnicity across various body habitats, including stool, were also detected in the Human Microbiome Project (HMP), where the greatest number of differentially distributed microbiota features (taxa, gene families, and metabolic pathways) with subject attributes were by race/ethnicity.^31^ Similarly, recent analyses of HMP and AG found specific taxa consistently associated with ethnicity across these two studies, and many of these taxa also have prior evidence of high heritability.^18^ Likewise, in this study, we found that race/ethnicity were significant determinants of the overall gut microbiota composition in both of the cohorts that we examined with black and white participants, and both studies showed a significant interaction between race/ethnicity and overweight/obese status. The oral and vaginal microbiomes have also been shown to correlate strongly with race/ethnicity.^32,33^ Interestingly, one study found that BMI was significantly correlated with vaginal microbiota composition among African but not European Americans.^34^ However, it cannot be ruled out that these microbiota-correlates with race/ethnicity may be driven by environmental factors instead of genes; we saw that dietary intake correlates with race/ethnicity in these cohorts and the gut microbiota-obesity relationship varied with diet. Prior studies that have compared the diets in black and white Americans, as well as in African Americans and rural Africans, detected differences in dietary intake between populations that correlated with differences in both fecal bacteria and its metabolites.^35,36^ Another recent study of over 1000 individuals concluded that genetic ancestry played a limited role in shaping the gut microbiome relative to the environment.^37^ However, this cohort was Israeli with limited racial and ethnic diversity, specifically very few black individuals.^37^ Thus, there is still a substantial gap in our understanding about how genetic ancestry shapes the gut microbiota, how the relationship between the gut microbiota and disease varies with genetic ancestry, and how these relationships differ with self-described race, which likely reflects diet, culture, socioeconomic status and other aspects of the environment more strongly than genetic ancestry.

Our work suggests that ethnic/racial differences across populations may explain at least some discordance in the nature of associations between the microbiota and obesity reported in different studies.^10,12^ Our observation that low alpha diversity may correlate with high BMI more consistently in NHWs than in black or Hispanic populations is consistent with many studies that are either exclusively or predominantly in NHW populations.^11–13^ Socioeconomic and health status correlate with race/ethnicity, and it is difficult to untangle these effects; AG had a fairly consistent association between lower alpha diversity and BMI across racial and ethnic groups, and the AG cohort is predominantly healthy, highly educated, and fairly wealthy individuals since participants pay to submit their microbiota samples. A recent meta-analysis and re-analysis of numerous studies of obesity and the gut microbiome included two Hispanic populations; one Hispanic Mexican-American cohort showed a trend towards lower alpha diversity with higher BMI, while a Columbian cohort was one of only two populations examined (among 10) that showed no trend towards such an association.^12^ Hispanic ethnicity is a genetic admixture of European, Native American and African that varies in proportion across the United States and across different countries.^38^ Interestingly, Colombia is one of the most genetically diverse group of Hispanics in Latin America with many individuals having a high proportion of African ancestry.^39^

Our observation that the association between *Prevotella* and BMI was most pronounced among blacks and Hispanics is consistent with a study in which three morbidly obese individuals, one black, one Hispanic, and one white (Krajmalnik–Brown, R. personal communication), were compared to three lean individuals,^14^ but such an association was also been noted in a morbidly obese European individual and was not seen in Hispanic Americans living on the Texas–Mexico border.^40,41^ The *Prevotella*-*Bacteroides* ratio was also seen to predict fat loss during a fiber-rich dietary intervention in Danish individuals, suggesting that the our results may be driven more by diet than by race or ethnicity.^40^ *Prevotella* has been shown to be enriched in prevalence and in diversity in non-Westernized societies, particularly *Prevotella copri*, which is near ubiquitous in non-Westernized populations and includes four distinct clades that tend to co-occur but are generally absent in Westernized populations.^42^ *Prevotella* is a complex genus that has been linked both to health and disease, and may interact with diet in complex ways.^19,24,42–44^ For example, *Prevotella*-rich microbiomes have been linked with a dietary-fiber induced improvement in glucose metabolism,^27^ but also with insulin resistance through the production of branched chained amino acids in the context of a high fat diet.^26^ While our analyses mainly focus on *Prevotella* and BMI, our meta-analysis of Obese Twins and GG imply that the relationship between the gut microbiota and obesity is not fully reflected by *Prevotella* relative abundance alone.

Our findings should be taken in the context of certain limitations. Only two of the studies included in our analyses included black individuals, and many of the included studies had small sample sizes or small numbers of non-whites. In order to fully understand whether there are distinct gut microbiota phenotypes of obesity and their associations with individual characteristics, much larger sample sizes are needed. We did not find evidence of strong confounding or interaction effects by smoking, but the studies had relatively small numbers of smokers. Diet was measured in different ways across the studies, and we were unable to examine race-based differences in the effects of diet in AG due to limited numbers of Hispanics and blacks. Likewise, it is important to understand the role of genetic risk for obesity and genetic ancestry in the microbiota-obesity relationship. While there are numerous cohorts with both genetic and microbiome information, most of these cohorts are also lacking in racial and ethnic diversity.^45^

Individuals with obesity vary in the extent of adiposity and also in the extent to which they suffer from other adverse health outcomes, including metabolic complications, such as hyperlipidemia, hypertension, glucose intolerance, and diabetes.^1,2^ Since obesity is a heterogeneous disease, and since several unique mechanisms by which the microbiota may influence obesity susceptibility have been proposed,^46^ it is also not surprising that there may be multiple distinct microbiota types that associate with obesity and that these may differ in prevalence in different cohorts. In fact, obese individuals with a low-diversity microbiota type have been characterized by more marked adiposity, greater inflammation, and poorer metabolic health compared to a high-diversity type.^11^ Another interesting question is whether different types of obesity-associated microbiotas may drive obesity by diverse mechanisms. Experiments in gnotobiotic or humanized mice that use different types of obesity-associated microbiotas as donor samples could help to determine whether different mechanisms may be at play.^6,14^

Since obesity rates are particularly high in non-white populations, our observation that the microbiota-obesity relationship may vary in unique ways by race/ethnicity underscores an urgent need to evaluate links between the microbiota and obesity in diverse populations, while simultaneously evaluating the role of race-associated factors, such as diet and socioeconomic status. Research about obesity and the gut microbiota has been dominated by studies focused on NHWs (e.g., ref. ^2,11,12^), but there is growing awareness about the importance of race, ethnicity and geography in determining the gut microbiota,^17,18^ and there are large racially and ethnically diverse cohorts that have more recently collected gut microbiome samples and will likely shed more light on these issues in the future.^47^ As various treatments for obesity vary greatly in efficacy across individuals, experiments that test the effects of weight loss treatments, e.g., various diets, in obese populations may benefit from a deeper understanding about different microbiota types and how they vary with race/ethnicity. This could facilitate personalized interventions, where the most effective strategies can be predicted based on the composition of the microbiota. The gut microbiota may also offer opportunities to treat or prevent obesity through personalized probiotics. However, microbiota-based interventions that do not take into account the differences in the gut microbiota or microbiota-disease relationships across diverse populations may be less effective or even have unintended adverse consequences.^17^

## Methods

### Datasets analyzed

The characteristics of individuals in the cohorts are reported in Table 1, and Supplemental Tables S1–S3. The Obese Twins sequenced data was previously published and made publicly available.^4^ In the Obese Twins dataset, we analyzed 270 gut samples from 152 people (Table 1). Most individuals had two samples, with an average time between samples of 57 ± 4 days.^4^ BMI changed minimally between the two samples. Diet information was collected via Food Frequency Questionnaires and was missing in some cases (Table 1). For regression models, we imputed smoking when missing (3.9%) using a two-step informed imputation based first on previous reported smoking (information was gathered at two time points within about 2 months^4^); when this was also missing, we used the R package mice^48^ to impute based on ancestry, age, and smoking in the family (twin or mother), since smoking was highly correlated among family members.

The GG 16 S rRNA data were from the samples described in Yatsunenko et al.^19^ However, whereas Yatsunenko et al. analyzed sequences from the V4 region of 16 S rRNA as sequenced on the Illumina platform, the data used here in conjunction with the Obese Twins data are publicly available but previously unpublished and represent sequences from the V2 region of rRNA that were sequenced on a 454 pyrosequencer with the same methods as used for Obese Twins,^4^ facilitating comparisons of the samples without having to correct for methodological differences used between studies. In the GG study, we analyzed samples from 491 individuals with sequences available from the V2 region. The data from the American Gut project, the Mexico City study, and EPOCH were previously described.^15,20,21^ We rarefied all of these datasets at the maximum level that allowed all (or most) of the samples to remain in the cohort; rarefaction levels for Obese Twins, AG, EPOCH and Mexico City were 1000/10,000/2537 and 1089 sequences per sample, respectively. We additionally rarefied at the minimum value across studies (1000) for a sensitivity analysis of the overall gut microbiota composition in order to control for differences between studies in terms of read depth.

Each study that we examined complied with all relevant ethical regulations and reported the specific details in the previously published manuscripts.^4,15,19–21^

### Exclusions

Some individuals from these cohorts were excluded from analyses. In AG, we excluded many individuals due with missing data or who feel outside of our inclusion criteria (Table S5). As in the AG manuscript, we excluded individuals with unrealistic values of BMI (weight and heights outside the range of 2.5–250 kg and 48–210 cm, respectively) and age (birth date after sampling date).^20^ We excluded young children (≤5 years) since their gut microbiota is quite distinct from that of adults, as well as those with unknown age. Only those who reported that they did not have IBD, IBS, or Autism and those who were from the USA or Canada were included in order to create a relatively homogeneous cohort of healthy participants. Since smoking status was missing in only 0.4% of individuals, it was imputed to the median (non-smoking). In EPOCH, black and Asian individuals were excluded due to very small numbers (*N* = 13 and 4, respectively). One individual was excluded from the Mexico City cohort due to missing BMI.

### Statistical methods

The characteristics of the cohorts were summarized by obesity status and stratified by race for Obese Twins and by race/ethnicity for the secondary cohorts and compared using Chi-squared tests or fisher exact tests for categorical variables and *t*-tests for continuous variables.

### Analyses of PC axes from meta-analysis of Obese Twins and GG

In order to test for significant differences in the coefficients of variation by obesity status across the PC axes, we used the R package cvequality.^49^ We tested the correlation between the PC1 axis and the alpha diversity measures using hierarchical linear regression models with PC1 as the outcome and the alpha diversity measures as the predictors, allowing for correlation by subject and family. We tested the correlation between the PC2 axis and percent abundance of the genus *Prevotella* in the same manner.

In order to examine the correlation between the principal coordinate axes with BMI, we used hierarchical linear regression models by race with BMI as the outcome and the principal coordinate axis, age and smoking status as predictors, allowing for correlation by subject within family.

### Analyses of determinants of overall gut microbiota composition

We used the Adonis function in the R vegan package^50^ to perform permutational ANOVA of the unweighted and weighted UniFrac^23^ distance matrices of each of the four studies analyzed. The predictors in these models varied according to the unique characteristics of each study. We included race (white or black), overweight/obese (ow/ob) status, their interaction, smoking status and age in the Obese Twins study; race/ethnicity (NHW, black, or Hispanic), ow/ob status, their interaction, smoking status, age and sex in AG; ethnicity (NHW or Hispanic), ow/ob status, their interaction, age and sex in EPOCH; and ow/ob status, age and sex in the Mexico City cohort. We performed this analysis using the maximum rarefaction level that allowed inclusion of all samples in each study, as well as at 1000 sequences per sample, which was the minimum rarefaction level across studies.

### Analyses of alpha diversity and BMI

In Obese Twins, we examined the relationship between alpha diversity and BMI using hierarchical linear regression models of BMI as a function of each of the standardized diversity measures (Shannon Diversity Index and PD Whole Tree) by race/ethnicity with correlation by subject within family. We controlled for age and smoking status. We used similar non-hierarchical linear regressions in the other three cohorts, controlling for the following potential confounding variables: sex, age, and smoking status in AG; sex and age in EPOCH; sex, age, HIV status and sexual practices (men who have sex with men versus not) in Mexico City.

### Analysis of Prevotella and BMI

We examined the relationship between *Prevotella* and BMI using the same methods as described above for alpha diversity. We modeled Prevotella using standardized relative abundance as well as the ratio of *Prevotella* relative abundance to the sum of the relative abundance of *Prevotella* and *Bacteroides*, as used previously.^15,40^ We used adjusted R^2^ to choose the better predictor of BMI.

### Evaluation of interactions with diet and smoking status

In the models of alpha diversity and *Prevotella*, described above, we checked for interactions with smoking and three dietary measures that we hypothesized may influence the gut microbiota-obesity relationship: fiber, fat, and meat intake. Smoking was only available for Obese Twins and AG; diet was additionally available in EPOCH. Diet was measured differently in AG compared to the other two studies; we chose the number of different types of plant consumed as the best marker for fiber; the only measure of meat and fat intake was a single measure called “high fat red meat.” In order to visualize significant interactions, we categorized alpha diversity and fiber as high (top quartile) and low-moderate (bottom 3 quartiles); plant intake as high (≥21) and low-moderate (≤20); high fat red meat as frequent (3–5 times per week or more) and low (<3 times per week). We categorized high versus low *Prevotella* relative abundance slightly differently in the two studies due to the vastly different distributions, and to create a reasonable distribution of data across the categories. In Obese Twins, we used the top tertile to define high *Prevotella*, and in AG, we used the mean.

### Evaluation of the confounding by smoking status

Associations between smoking and (1) alpha diversity, (2) *Prevotella*, and (3) BMI, were examined using regressions similar to those described for alpha diversity, but with alpha diversity/*Prevotella* / BMI as the outcomes and smoking status (non-imputed) as the primary exposure. We controlled for age and race in Obese Twins, and age, race/ethnicity, and sex in AG.

For these analyses, we used R v3.5.0,^49^ SAS V9.4 (SAS Institute Inc., Cary, North Carolina), and Qiime v1.9.1.^51^ We considered two-sided *p*-values of 0.05 or less as statistically significant.

### Reporting summary

Further information on research design is available in the Nature Research Reporting Summary linked to this article.

## Supplementary information

Supplementary Material.

Reporting Summary checklist

## Data Availability

The data from the Obese Twins and AG data are available online (https://zenodo.org/record/840333 and ftp://ftp.microbio.me/AmericanGut/). The EPOCH data are available from the European Bioinformatics Institute (ERP112799) and Mexico City data are in the Sequence Read Archive (PRJNA344791). The GG DNA sequences can be found in the MG-RAST server (mgp401).

## Supplementary information

**Supplementary information** accompanies the paper on the *npj Biofilms and Microbiomes* website (10.1038/s41522-019-0091-8).

## Code availability

All code used in analyses are available by request from the corresponding author.
