# Supplementary material for: Gut microbiota phenotypes of obesity
Source: NPJ Biofilms Microbiomes. 2019 Jul 1;5:18. doi: 10.1038/s41522-019-0091-8 (PMC6603011; doi:10.1038/s41522-019-0091-8)
Supplement: Supplementary file 1 — Supplementary Material. [file 41522_2019_91_MOESM1_ESM.pdf]

Supplementary Figures

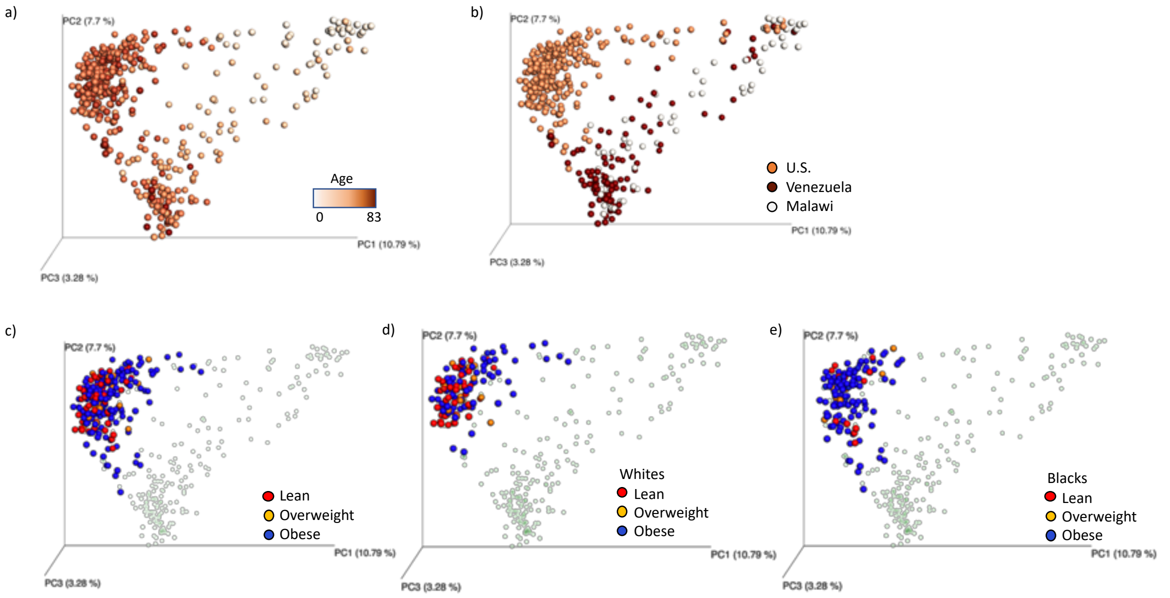

Supplementary Figure 1: Meta-analysis of 16S rRNA data from Obese Twins and GG using Unweighted UniFrac. PCoA plot with points colored a) across an age gradient, b) by country, c) by obesity status overall, d) among whites and e) among blacks.

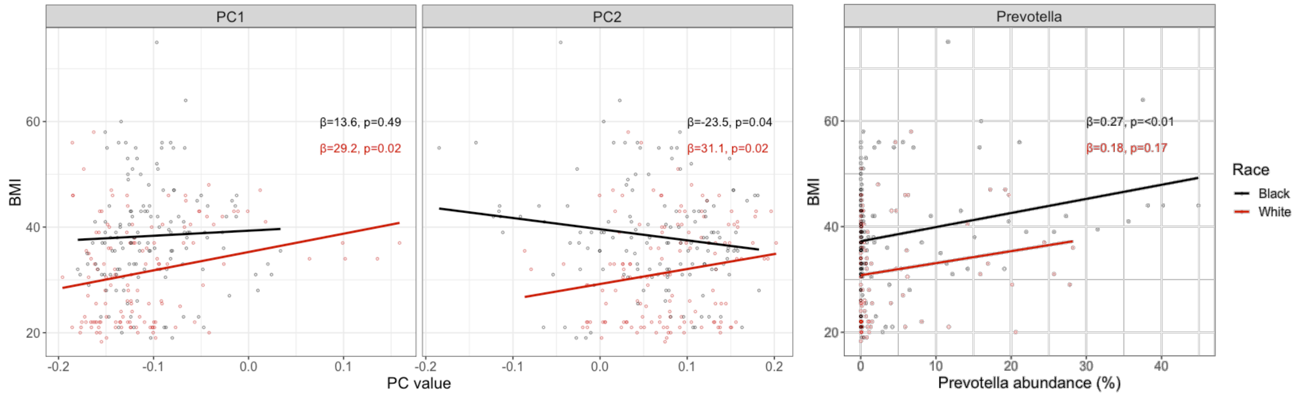

Supplementary Figure 2. a) Plots of the PC axis values against BMI in the Obese Twins study. Obese outliers were noted on these two PCoA axes, with higher prevalence of white outliers on PC1 and black outliers on PC2. Regression models support a difference in the association between BMI and these PC values by race: BMI increases significantly with PC1 for whites but not for blacks (left); BMI increases with PC2 axis for whites but decreases with PC2 for blacks (right). PC1 is inversely correlated with alpha diversity (p-value<0.01 for PD, Shannon and observed species); PC2 is inversely correlated with *Prevotella* relative abundance (p-value<0.01). However, the relationship between BMI and relative abundance of *Prevotella* (b) is much less dependent on race, although it is only significantly associated for blacks.

Supplementary Tables

Supplementary Table 1. Characteristics of the AG cohort by race / ethnicity.

	**Black**	**Hispanic**	**NHW**	**p-value**
**N**	**63**	**188**	**4784**	
Age	22 (34.9)	107 (56.9)	2219 (46.4)	0.003
Male Sex	41.44 (14.91	42.60 (16.25	47.81 (15.65	<0.001
BMI Category				<0.001
Lean	16 (25.4)	94 (50.0)	3021 (63.1)	
Overweight	21 (33.3)	56 (29.8)	1252 (26.2)	
Obese	26 (41.3)	38 (20.2)	511 (10.7)	
BMI	29.12 (6.31)	26.43 (5.56)	24.68 (4.79)	<0.001
Smoker	12 (19.0)	16 (8.5)	307 (6.4)	<0.001
**Diet**				
Vegetable frequency				<0.001
Never	0 (0.0)	2 (1.1)	14 (0.3)	
Rarely - less than once week	7 (11.1)	7 (3.7)	104 (2.2)	
Occasionally - 1-2 times week	13 (20.6)	34 (18.1)	321 (6.7)	
Regularly - 3-5 times week	11 (17.5)	65 (34.6)	1193 (24.9)	
Daily	12 (19.0)	45 (23.9)	1409 (29.5)	
Unknown	20 (31.7)	35 (18.6)	1743 (36.4)	
Number of different types of plants				<0.001
Less than 5	8 (12.7)	25 (13.3)	252 (5.3)	
6 to 10	15 (23.8)	56 (29.8)	870 (18.2)	
11 to 20	11 (17.5)	55 (29.3)	1268 (26.5)	
21 to 30	4 (6.3)	12 (6.4)	694 (14.5)	
More than 30	3 (4.8)	0 (0.0)	522 (10.9)	
Unknown	22 (34.9)	40 (21.3)	1178 (24.6)	
High fat red meat frequency				0.726
Never	7 (11.1)	25 (13.3)	600 (12.5)	
Rarely - less than once week	16 (25.4)	57 (30.3)	1261 (26.4)	
Occasionally - 1-2 times week	13 (20.6)	49 (26.1)	827 (17.3)	
Regularly - 3-5 times week	6 (9.5)	19 (10.1)	298 (6.2)	
Daily	1 (1.6)	2 (1.1)	39 (0.8)	
Unknown	20 (31.7)	36 (19.1)	1759 (36.8)	
**Markers of socioeconomic status**				
Level of education				<0.001
Did not complete high school	2 (3.2)	2 (1.1)	27 (0.6)	
High School or GED equilivant	4 (6.3)	15 (8.0)	69 (1.4)	
Some college or technical school	8 (12.7)	39 (20.7)	291 (6.1)	
Associate's degree	3 (4.8)	11 (5.9)	110 (2.3)	
Bachelor's degree	9 (14.3)	33 (17.6)	881 (18.4)	
Some graduate school or professional	5 (7.9)	17 (9.0)	249 (5.2)	
Graduate or Professional degree	12 (19.0)	33 (17.6)	1410 (29.5)	
Unknown	20 (31.7)	38 (20.2)	1747 (36.5)	

Supplementary Table 2. Characteristics of the EPOCH cohort ethnicity.

	Hispanic	NHW	p-value
**N**	**43**	**59**	
Age	15.66 (1.05)	15.83 (1.00)	0.406
Male sex	24 (55.8)	33 (55.9)	1
BMI z-score	0.56 (1.11)	0.44 (1.07)	0.564
BMI category			0.615
Lean	28 (65.1)	42 (71.2)	
Obese	9 (20.9)	8 (13.6)	
Overweight	6 (14.0)	9 (15.3)	
**Diet**			
Kilocalories	1615 (586)	1733 (694)	0.372
% Carbohydrates	48 (7.0)	48 (7.0)	0.788
% Fat	38 (5.0)	38 (6.0)	0.983
% Protein	16 (2.0)	15 (2.0)	0.577
Meat servings	1.19 (0.70)	1.05 (0.67)	0.336
Vegetable servings	1.80 (1.12)	1.56 (1.01)	0.266
Dark green cruciferous	0.52 (0.52)	0.44 (0.47)	0.382
Dark yellow	0.29 (0.43)	0.23 (0.29)	0.426
Other vegetables	0.35 (0.33)	0.33 (0.36)	0.703
Sweets	1.21 (1.08)	1.54 (1.36)	0.209
Total fiber	12.97 (5.54)	12.91 (5.82)	0.964
Soluble fiber	4.55 (1.88)	4.51 (1.91)	0.922
Insolble fiber	8.30 (3.72)	8.28 (3.92)	0.977
**Markers of socioeconomic status**			
Parental education			0.018
High school, GED or less	9 (20.9)	5 (8.5)	
Associate degree or some college	17 (39.5)	13 (22.0)	
Bachelor's degree	11 (25.6)	21 (35.6)	
Graduate degree	6 (14.0)	20 (33.9)	
Household income			0.034
$16,000-34,999	2 (4.7)	2 (3.4)	
$35,000-49,999	11 (25.6)	4 (6.8)	
$50,000-74,999	11 (25.6)	10 (16.9)	
$75,000-99,999	7 (16.3)	11 (18.6)	
$100,000 or more	12 (27.9)	29 (49.2)	
Unknown	0 (0.0)	3 (5.1)	

Supplementary Table 3. Characteristics of the Mexico City cohort.

	Hispanic
**N**	**42**
Age	40.19 (10.14)
Male Sex	34 (81.0)
BMI	24.29
BMI Category	
Lean	26 (61.9)
Overweight	12 (28.6)
Obese	4 (9.5)
Smoking status	
Unknown	42 (100.0)
HIV status	
HIV-	9 (21.4)
HIV+	33 (78.6)
Men who have sex with men	
No	6 (14.3)
Yes	20 (47.6)
Unknown	16 (38.1)

Supplementary Table 4. This table shows the association between individual characteristics and the overall gut microbiota composition in the 4 studies examined, using Permutational ANOVA of the unweighted and weighted UniFrac distance matrices. The first columns show the results using the rarefaction levels used throughout the analyses in the manuscript, as well as at 1000 sequences per sample, which is the minimum value used across studies (Obese Twins). The R^2 values indicate how much of the variation in the gut microbiota composition is explained by each effect, with the associated p-value. The individual characteristics examined varied by study and reflect the unique characteristics of each cohort.

		Varying rarefaction by study				Rarefaction of 1000			
		Unweighted UniFrac		Weighted UniFrac		Unweighted UniFrac		Weighted UniFrac	
Study	Effect	R^2	P-value	R^2	P-value	R^2	P-value	R^2	P-value
Obese Twins	Race	1.2%	0.001	2.6%	0.001	1.2%	0.001	2.6%	0.001
Rarefaction	Overweight / obese	0.8%	0.001	1.4%	0.008	0.8%	0.001	1.4%	0.008
1000	Smoking (imputed)	0.6%	0.001	1.4%	0.009	0.6%	0.001	1.4%	0.009
	Age	0.7%	0.001	1.3%	0.013	0.7%	0.001	1.3%	0.013
	Race*Overweight / obese	0.5%	0.012	0.4%	0.299	0.5%	0.012	0.4%	0.299
AG	Race	0.1%	0.001	0.4%	0.001	0.1%	0.001	0.3%	0.001
Rarefaction	Overweight / obese	0.2%	0.001	0.1%	0.003	0.2%	0.001	0.1%	0.005
10,000	Smoking (imputed)	0.1%	0.001	0.1%	0.013	0.1%	0.001	0.1%	0.001
	Sex	0.3%	0.001	0.5%	0.001	0.2%	0.001	0.4%	0.001
	Age	0.6%	0.001	0.3%	0.001	0.5%	0.001	0.4%	0.001
	Race*Overweight / obese	0.06%	0.001	0.1%	0.016	0.05%	0.002	0.1%	0.006
EPOCH	Ethnicity	0.8%	0.774	1.7%	0.123	0.7%	0.882	1.7%	0.092
Rarefaction	Overweight / obese	1.2%	0.132	0.6%	0.684	1.1%	0.302	0.6%	0.713
2537	Sex	1.5%	0.046	0.7%	0.601	1.5%	0.034	0.7%	0.599
	Age	1.1%	0.293	0.9%	0.448	1.1%	0.235	1.0%	0.369
	Ethnicity*Overweight / obese	1.1%	0.286	1.3%	0.203	1.1%	0.234	1.2%	0.261
Mexico Cty	Overweight / obese	3.6%	0.035	3.4%	0.171	3.5%	0.034	3.2%	0.194
Rarefaction	HIV Status	3.9%	0.011	1.4%	0.612	4.2%	0.01	1.5%	0.553
1089	MSM	2.4%	0.339	3.5%	0.160	2.8%	0.156	3.4%	0.152
	Sex	3.7%	0.021	10.3%	0.006	3.9%	0.014	10.8%	0.002
	Age	3.1%	0.080	4.5%	0.088	2.7%	0.177	4.7%	0.091

Supplementary Table 5. This table shows the exclusion criteria from the AG cohort, as well as the racial / ethnic distribution of the remaining samples.

Inclusion	N	Black	Hispanic	White	Other
AG 100NT Samples	17,876	124	399	15,041	2,312
Stool	15,158	106	360	13,138	1,554
Race: White, Black, Hispanic	13,604	106	360	13,138	NA
BMI within realistic range	13,216	93	327	12,555	NA
BMI category: Normal, Overweight, or Obese	11,860	88	292	11,480	NA
No dx of IBD/IBS/ASD	9,482	78	258	9,146	NA
Country: USA or Canada	7,050	77	239	6,734	NA
Known age>5	6,853	73	227	6,553	NA
Rarefied at 10000 sequences / sample	5,035	63	188	4,784	NA
